# Learning Curve Complications and Management Strategies in Unilateral Biportal Endoscopic Spine Surgery: A Case Series

**DOI:** 10.7759/cureus.98479

**Published:** 2025-12-04

**Authors:** Alhareth Maaya, Jin Hwa Eum

**Affiliations:** 1 Neurosurgery, Ain Al Khaleej Hospital, Al Ain, ARE

**Keywords:** biportal endoscopic spine surgery, complication, csf leakage, minimal invasive surgery, spinal cord

## Abstract

This case series aims to highlight complications encountered during the early learning curve of unilateral biportal endoscopic (UBE) spine surgery, emphasizing challenges in managing pseudomeningoceles, operatively encountered conjoined nerve roots, and the unique complication of selective sensory deficits following an unexpected cranial direction of the C6 nerve root. The purpose is to emphasize the ongoing learning process required to optimize patient outcomes. Three cases were retrospectively reviewed. Case 1 describes a 36-year-old man with L5/S1 disc prolapse who underwent left UBE discectomy complicated by a major dural tear managed intraoperatively with glue but later required two additional surgeries for dural repair and continues to experience neuropathic pain. Case 2 involves a 35-year-old woman who underwent left UBE for L5/S1 discectomy and subsequently developed a small pseudomeningocele with intraoperatively encountered conjoined nerve roots, with persistent neuropathic pain despite conservative treatment. Case 3 involves a 45-year-old woman who underwent right C5/C6 UBE discectomy, during which an unexpected cranial orientation of the C6 nerve root was noted, resulting in postoperative selective sensory deficits that improved with conservative management over six weeks. This series focuses on complications encountered during the early phase of UBE surgery, where Case 1 illustrates the difficulty of managing large dural tears, Case 2 highlights the implications of conjoined nerve roots and pseudomeningocele-related neuropathic symptoms, and Case 3 describes atypical selective sensory deficits resulting from an aberrant C6 nerve root trajectory. An algorithmic representation of management strategies is included to assist decision-making for surgeons in the early stages of adopting UBE techniques. The findings underscore that the early learning curve of UBE spine surgery presents several unique challenges, particularly involving dural tears, anatomical variations such as conjoined nerve roots, and unexpected nerve root trajectories, and recognizing these issues while implementing appropriate management strategies can improve patient outcomes and support novice surgeons in refining their operative skills.

## Introduction

Spine surgery has been moving toward minimally invasive techniques, and one such approach is unilateral biportal endoscopic (UBE) spine surgery. The increasing success of this novel system necessitates an analysis of its early learning curve and the potential challenges encountered during its initial implementation. The unilateral biportal endoscopic (UBE) spinal surgery is a hybrid technique that combines elements of both open and endoscopic spinal surgery [1,2]. This newer approach has become very popular and is in high demand because it allows the use of standard surgical instruments such as curettes, Kerrison punches, osteotomes, high-speed drills, and forceps, making it a cost-effective option. In comparison to uniportal full endoscopic surgery, UBE offers the advantage of a shorter learning curve [3,4].

There is growing evidence in the literature supporting UBE discectomy as a suitable replacement for traditional laminotomy and microdiscectomy in the treatment of lumbar disc herniation (LDH) [1,2,5]. Studies have demonstrated that single-level UBE discectomy provides equivalent pain control, functional improvement, and patient satisfaction while also offering additional advantages such as minimal blood loss, reduced hospital stay, and less postoperative back pain compared to microdiscectomy [6,7]. Despite the well-documented success of UBE discectomy in both clinical and radiological outcomes, it remains essential to acknowledge the potential complications associated with the technique. As with both uniportal endoscopic spinal surgery and conventional open microscopic approaches, UBE procedures carry inherent risks that must be carefully assessed and managed.

The title of this paper, "Learning Curve Complications and Management Strategies in Unilateral Biportal Endoscopic Spine Surgery: A Case Series," reflects our commitment to highlighting the difficulties encountered during the early implementation of UBE spine surgery. This case series addresses complex complications seen during the initial learning curve, particularly incidental durotomy, operatively encountered conjoined nerve roots, and the rare complication of selective sensory deficits arising from an unexpected cranial orientation of the C6 nerve root [2,5]. The main purpose of this research is to delineate the dilemmas faced by spine surgeons during the early stages of adopting UBE into their practice. Through real-world clinical scenarios, we aim to provide valuable insights into the nuanced management strategies required to address these complications and improve patient outcomes.

As the popularity of minimally invasive spine surgery continues to grow, a comprehensive understanding of UBE intricacies and potential pitfalls becomes essential for surgeons striving to refine their skills and ensure high-quality patient care. Even during the initial evaluation of patients, surgical teams must anticipate challenges such as managing incidental durotomy, encountering conjoined nerve roots, and addressing unusual complications such as selective sensory deficits [1]. Conducting detailed analyses of actual clinical cases allows us to promote a clearer understanding of the UBE learning curve. This case series is expected to equip practitioners with meaningful knowledge that can support complication management, enhance the safe adoption of this technique, benefit patients, and contribute to the ongoing advancement of spine surgery.

This study involved a retrospective review of patients who underwent unilateral biportal endoscopic (UBE) spine surgery. Patient selection focused specifically on individuals who experienced complications following their UBE procedure. Inclusion criteria required a documented record of postoperative or intraoperative complications directly related to UBE surgery. Cases were selected from the operating surgeon's clinical practice to ensure a representative cross-section of complications commonly encountered during the early learning curve of the technique.

Data collection involved a thorough review of all available medical records, including preoperative evaluations, intraoperative documentation, postoperative progress notes, and follow-up assessments. Collected data included demographic information, medical history, preoperative diagnosis, operative details, and the nature of postoperative complications. These parameters allowed for a comprehensive understanding of each case and facilitated detailed analysis of the complications and management strategies relevant to the early stages of UBE adoption.

## Case presentation

Case 1: Treatment of major dural tear and persistent neuropathic pain

**Figure 1:**
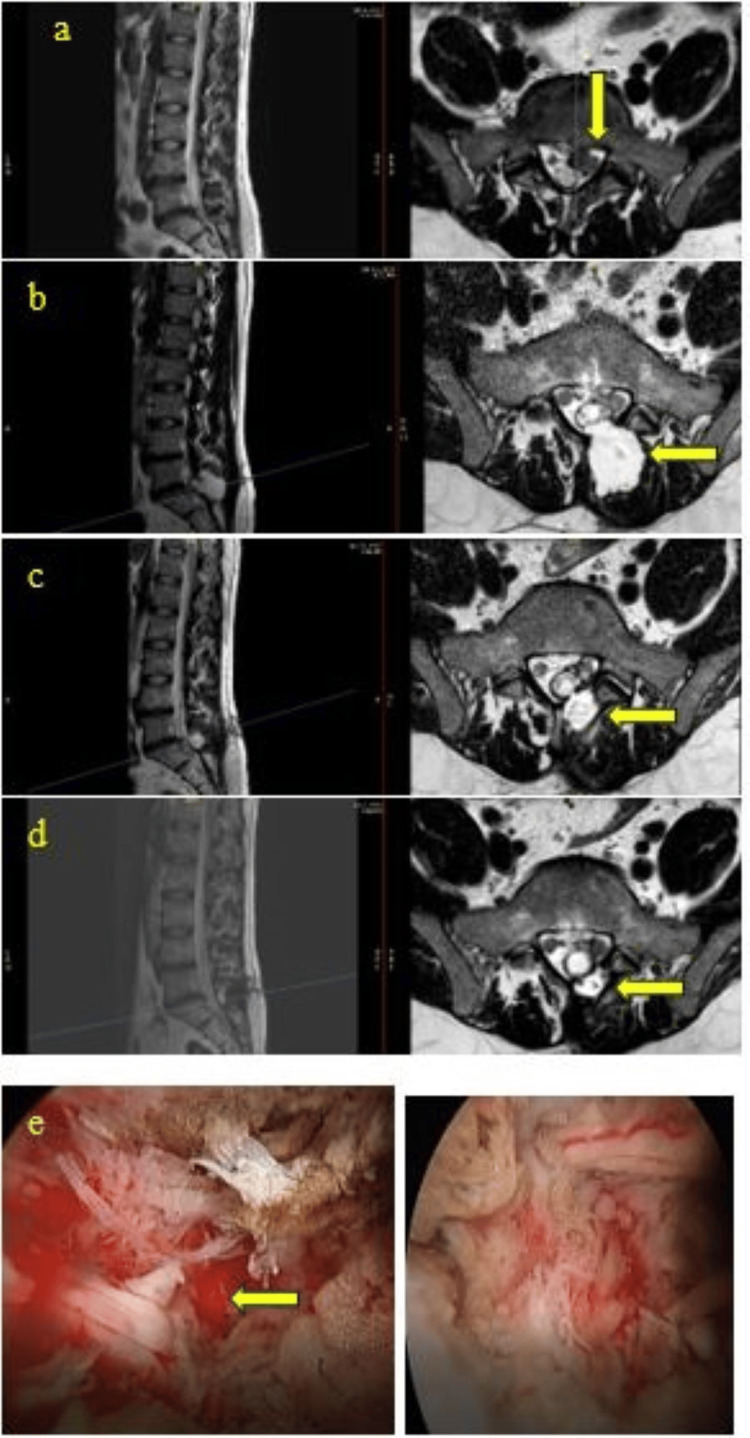
Dural Tears and Repeated Surgeries (a) Preoperative axial and sagittal T2 MRI of the left L5/S1 disc herniation compressing the nerve root (yellow arrow). (b) Postoperative axial and sagittal T2 MRI showing a large pseudomeningocele (yellow arrow). (c) Axial and sagittal T2 MRI following the primary surgical dural repair showing a decrease in pseudomeningocele but an incomplete repair with CSF collection (yellow arrow). (d) Axial and sagittal T2 MRI after the second surgical repair showing a good and successful repair (yellow arrow). (e) Intraoperative images showing a significant durotomy, with nerve roots seen outside the dural sac (yellow arrow) CSF: cerebrospinal fluid

A 36-year-old man presented with a disc prolapse at the L5/S1 level and underwent a left unilateral biportal endoscopic (UBE) discectomy. The surgery was complicated by a large dural tear, which was managed intraoperatively with glue. Postoperatively, the patient was placed on complete bed rest for three days, and at the time of discharge one week later, there was no cerebrospinal fluid (CSF) leak or bulge. Two weeks later, however, the patient presented to the emergency department with fever and cough and was diagnosed with influenza pneumonia. During this episode, a bulge developed without an associated CSF leak, and he was discharged after one week. Despite conservative measures, including rest and aspiration, his symptoms persisted. The patient subsequently underwent two additional surgeries at outside institutions to repair the dural tear, yet he continues to experience neuropathic pain. This case demonstrates the low satisfaction rate associated with repeated operations for major dural tears and underscores the difficulties of managing dural complications during the early learning curve of UBE spine surgery. Notably, this surgery represented the ninth case in the primary author's learning curve, reflecting the higher likelihood of encountering durotomies early in UBE practice, particularly major ones (Figure 1).

Case 2: Persistent neuropathic pain, small pseudomeningocele, and postoperative CSF leak management

**Figure 2:**
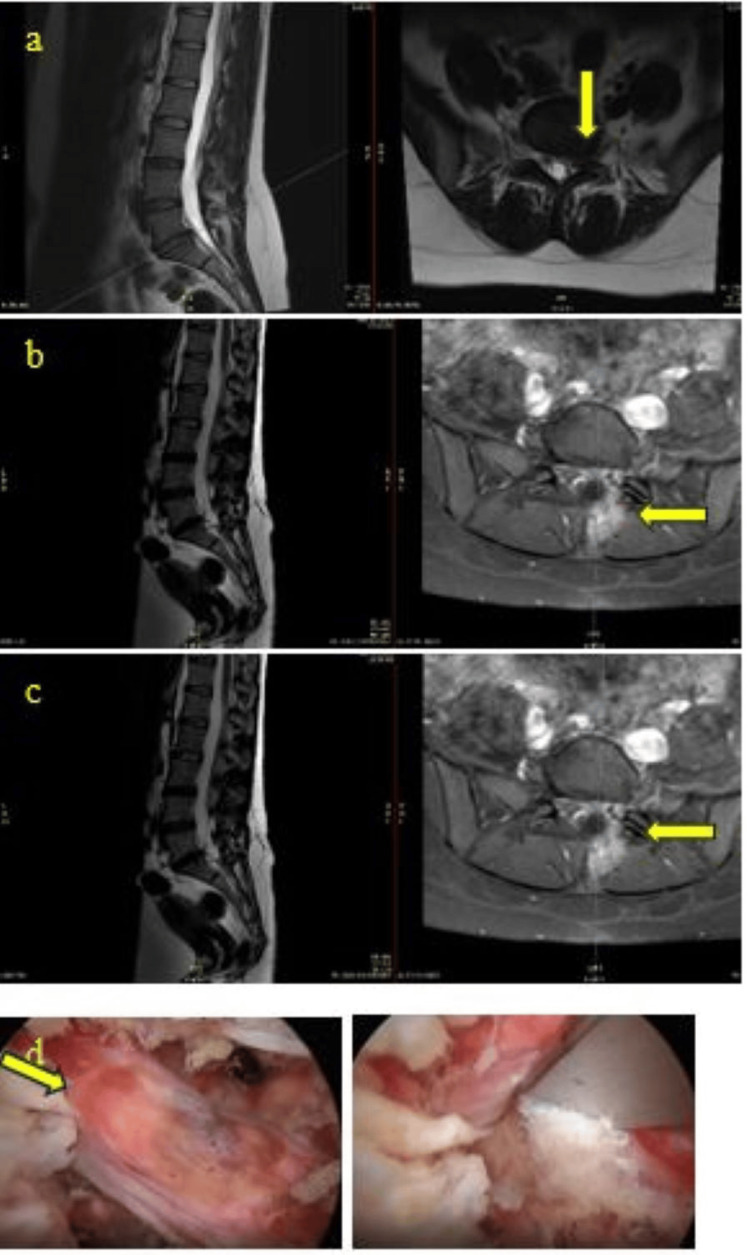
Persistent Neuropathic Pain (a) Preoperative axial and sagittal T2 MRI demonstrating left L5/S1 disc herniation, which compresses the nerve root (yellow arrow). (b) Postoperatively, axial and sagittal T2 MRI showing the left conjoined nerve root and a small pseudomeningocele (yellow arrow). (c) Axial and sagittal T2 MRI at an 18-month follow-up postoperatively, indicating the left conjoined nerve root and an unchanged small pseudomeningocele (yellow arrow). (d) Intraoperative images delineate conjoined nerve roots (yellow arrow)

A 35-year-old woman underwent left unilateral biportal endoscopic (UBE) L5/S1 discectomy. Postoperatively, she developed a small pseudomeningocele, and intraoperatively, conjoined nerve roots were identified, although there was no evidence of durotomy. Attempts to perform a discectomy through the axilla of the nerve root and between the conjoined nerve roots were made without causing a durotomy. On the first postoperative day, the patient developed a CSF leak, which resolved with rest and a pressure dressing. By the time of discharge one week later, there was no evidence of persistent CSF leakage; however, she continued to experience neuropathic pain despite conservative treatment. This case highlights the challenges of pain management associated with small pseudomeningoceles and the complexity of surgically addressing conjoined nerve roots during UBE spine surgery. The intertwined nature of the conjoined roots complicates both intraoperative maneuverability and postoperative care. Interestingly, the patient initially underwent MRI at an external facility before having surgery at our center, but the preoperative MRI did not clearly reveal the presence of conjoined roots, likely due to poor-quality imaging or disc compression. This emphasizes the importance of meticulous preoperative imaging review and the need for heightened awareness of potential complications such as postoperative CSF leaks when managing similar cases (Figure 2).

Case 3: Sensory disturbances and cranial atypical direction of the C6 nerve root

**Figure 3:**
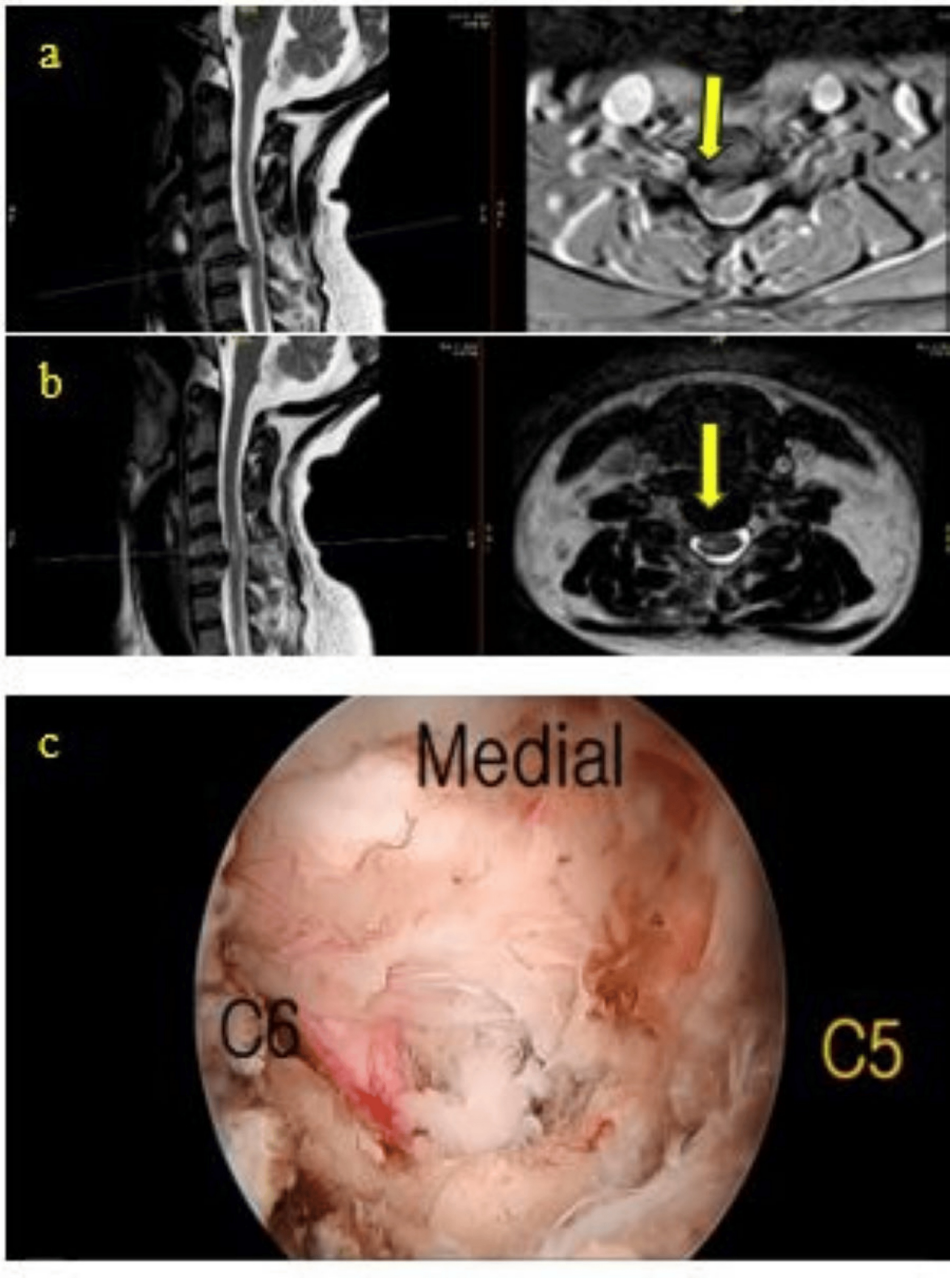
Unique Sensory Deficits (a) Preoperatively, axial and sagittal T2 MRI showing right C5/C6 disc herniation provoking the compression of the root and spinal cord (yellow arrow). (b) MRI in the axial and sagittal T2 sequences of the postoperative period showing the decompression of the nerve root and spinal cord with a hyperintense signal on the right anterolateral aspect (yellow arrow). (c) The intraoperative picture displays a cervical nerve root with an atypical cranial direction

A 45-year-old woman underwent right C5/C6 unilateral biportal endoscopic (UBE) discectomy, during which an unexpected cranial orientation of the C6 nerve root was identified. Both cranial and caudal discectomy and foraminotomy were performed to sufficiently decompress the nerve root. Postoperatively, the patient's visual analog scale (VAS) score improved significantly from 10/10 to 1/10, and she was discharged the following day. One week later, she reported an inability to sense temperature changes in the left lower limb extending up to the umbilicus during bathing, although she exhibited no motor deficits or radicular pain. MRI demonstrated a hyperintense signal in the cervical region corresponding to the right spinothalamic tract. Her sensory symptoms gradually improved over six weeks with conservative management. This case illustrates a rare example of partial sensory deficits resulting from an unusual cranial course of the C6 nerve root during UBE spine surgery. The patient's recovery highlights the nervous system's capacity for adaptation following unexpected intraoperative findings and emphasizes the importance of anticipating anatomical variations during UBE procedures (Figure 3).

## Discussion

The case series emphasizes the subtleties and challenges of the initial learning phase of unilateral biportal endoscopic (UBE) spine surgery. The unusual problems encountered in these cases illustrate the importance of constant learning, careful assessment, and individualized decision-making to ensure that patients benefit from this developing surgical technique. The complications observed in UBE spine surgery span a wide range, beginning with the significant issue of dural tears and the potential need for repeated surgeries, as demonstrated in Case 1. The challenges of managing large dural tears at the outset of UBE procedures were evident in the 36-year-old male patient who continued to experience neuropathic pain despite multiple revision surgeries. This underscores the necessity of perfecting intraoperative repair techniques and maintaining diligent postoperative follow-up. A broader examination of dural tear incidence across surgical techniques reveals that the rate of dural tears is significantly lower in minimally invasive surgery (MIS) discectomy than in open surgery, supporting the benefits of MIS approaches in reducing this complication. In the context of revision surgery, the MIS group also shows significantly fewer dural tears compared to the open surgery group (4.2% versus 19.4%, p=0.028), although no significant differences are noted in TLIF or decompression procedures such as laminectomy or laminotomy [8].

Dural tears are considered the most common complication in UBE, with an incidence of 1.5%-5.8%. Multiple risk factors contribute to this complication, including instrument- or radiofrequency-related injury, adhesions within the spinal canal, giant disc fragments, and lax dura mater. The careful exploration of the meningovertebral ligament is essential, and the management strategy varies based on tear size, with small tears generally treated with bed rest and observation, while larger defects require open repair [9]. The durotomy rate also varies significantly with surgeon experience; rates range from 1% to 10% among less-experienced surgeons compared to approximately 0.1% among surgeons who have performed thousands of cases, as noted in a recent survey of 97 spine surgeons. In that study, 20.4% of surgeons accounted for 70% of reported durotomies, with only 29% having more than 10-15 years of experience and 48% having only 1-5 years of spinal endoscopy experience. These findings imply that the true incidence of durotomies may be underreported among early-career endoscopic spine surgeons [10]. The risks associated with uniportal endoscopic spine surgery (ESS), including incidental durotomy, are similarly well-documented, with an overall incidence ranging from 0% to 8.6% in prior studies. Variability in these rates highlights the importance of surgeon experience and meticulous technique, particularly because incidental durotomy can require conversion to open surgery. This reinforces the need for cautious planning and the precise execution of endoscopic interventions to prevent complications, minimize invasiveness, and achieve optimal outcomes [11].

Persistent neuropathic pain, as illustrated in Case 2, further demonstrates the complexity of managing UBE-related complications. The 35-year-old female patient continued to experience neuropathic pain associated with a small pseudomeningocele despite conservative treatment, emphasizing the difficulty of managing postoperative symptoms when conjoined nerve roots are encountered intraoperatively. Conjoined lumbosacral nerve roots, defined as two adjacent roots sharing a common dural envelope at some point along their course, have a reported incidence of 2%-17.3% in lumbar spine imaging studies, with cadaveric studies reporting up to 30%. In the specific context of surgical practice, the incidence reported was 5.8%, most frequently involving the L5-S1 roots in 69% of cases and L4-L5 in 31%. Patients often present with claudication, with or without radiculopathy [12]. Even with advancements in imaging, including magnetic resonance (MR) neurography, identifying conjoined nerve roots can remain challenging, particularly when masking factors such as large disc herniation or severe spinal stenosis are present [13]. During surgery, these nerve roots may be difficult to identify as extruded disc fragments can hide within the secondary axilla. Conjoined roots are also less mobile and more difficult to retract, complicating disc removal. Extending the interlaminar space proximally can improve visualization and help safely retract roots for procedures such as herniotomy or nucleotomy. Surgeons must remain vigilant for nerve root variations and ensure adequate decompression proximally to create sufficient operative space and minimize nerve injury risk [14].

Unique sensory deficits, as described in Case 3, further underline the complexities of UBE spine surgery. The unexpected cranial direction of the C6 nerve root contributed to the selective loss of pain and temperature sensation, demonstrating the need for flexible postoperative planning when unanticipated anatomical findings arise. Literature exploring the angle between cervical rootlets and the spinal cord shows a gradual decrease caudally, with dynamic variations during neck movement from flexion to extension [15]. Notably, the smallest angle between the nerve root and the lateral margin of the dura is reported for the C6 nerve root at 50.9°±6.4°, highlighting meaningful anatomical variation that may influence operative navigation [16]. Additional anatomical details describe the funnel-shaped morphology of the intervertebral foramina, the relationship of cervical nerve roots to the vertebral column, the invagination of ventral and dorsal roots creating depressions in the dural sac, and specific angulation patterns as the roots exit through the dura [17]. These details are essential for surgeons performing UBE procedures, as an appreciation of such variations can support precise intraoperative navigation and reduce complications.

The learning curve plays a central role in shaping outcomes, as this case series reflects the inherent difficulties surgeons encounter when adopting UBE spine surgery, particularly those related to dural repair and unexpected anatomical variations. The emphasis on continual learning underscores the evolving nature of mastering UBE techniques and supports the view that lifelong education and refinement of surgical skills are crucial. The persistent neuropathic pain observed in these cases reinforces the need for tailored strategies in postoperative management, integrating both surgical and nonsurgical approaches, along with comprehensive patient education. Furthermore, the recognition of distinct complications such as unusual nerve root orientation stresses the importance of adaptive clinical reasoning and flexible management pathways. Clinically, these cases are highly relevant for surgeons practicing UBE techniques, as they provide valuable insights into predicting and managing potential complications. Future directions involve refining training programs, enhancing the exchange of surgical experiences within the spine community, and continuously modifying surgical protocols as new evidence and case experiences emerge.

## Conclusions

In summary, the steep learning curve associated with unilateral biportal endoscopic spine surgery needs a strong patient to go through the learning process. The identification of specific issues, such as unexpected anatomical variations, allows surgeons to hone their skills and adaptively change strategies to attain good patient outcomes. This study is a valuable part of the overall knowledge of UBE spine surgery that makes the practitioners more skilled and patient-centered.
